# Fulminant hepatic failure due to metastatic choroidal melanoma

**Published:** 2017

**Authors:** Emmanuel Escobar-Valdivia, Roberto Monreal-Robles, Guillermo Delgado-García, Badir Hernández-Velazquez

**Affiliations:** 1 Department of Internal Medicine, Autonomous University of Nuevo Leon, Monterrey, Mexico.; 2 Division of Gastroenterology, University Hospital, Autonomous University of Nuevo León.

**Keywords:** Fulminant liver failure, Acute liver failure, Melanoma, Metastatic melanoma, Choroidal melanoma

## Abstract

**Background:**

Acute liver failure (ALF) as a consequence of metastatic disease is extremely uncommon. The liver is the most commonly affected organ by metastatic disease, but only a few cases of ALF in the setting of metastatic choroidal melanoma have been reported.

**Case Presentation:**

We describe the case of a 47-year-old man with right upper quadrant pain, progressive jaundice, and unintentional weight loss. He also reported that he had experienced reduced left visual acuity which progressed to blindness over 2 months. On physical examination, we found a pigmented scleral lesion in the left eye. He had a coagulopathy and**,** during his hospital stay, he also developed encephalopathy. The diagnosis of ALF was therefore established and was later attributed to metastatic uveal melanoma. In addition, we briefly review the relevant literature.

**Conclusion:**

Liver metastasis should be kept in mind when assessing abnormal liver function tests in patients with uveal malignant melanoma.

**A**cute liver failure (ALF) as a consequence of metastatic disease is extremely uncommon. In a 17-year period, the Liver Failure Unit at King's College Hospital reported a prevalence of ALF secondary to metastatic disease of 0.44%; none due to malignant melanoma (1). Even though the liver is the most common affected organ by metastatic disease, only a few cases of ALF in the setting of metastatic choroidal melanoma have been reported (2-6). Here, we report an additional case and then we briefly review the relevant literature.

## Case presentation

A 47-year-old man presented to the emergency department with a 2-month history of right upper quadrant pain, progressive jaundice and unintentional weight loss (about 15 kg). On further questioning, he revealed that he had experienced reduced left visual acuity which progressed to blindness over 2 months. A year earlier, he also developed a pigmented scleral lesion in the ipsilateral eye and was not seen by a health care provider then. The physical examination was notable for jaundice and tender hepatomegaly (18 cm by palpation). He had heterochromia iridis. Three conjunctival lesions with superficial vascularization were found in the same eye, one of them producing uveal ectropionization. Pupillary areflexia was alike present. On fundoscopic examination, pale optic disc with hemorrhages in the temporal arcades and superior retina were detected.

Pigmentary changes were likewise detected in the inferior retina. Initial laboratory workup showed lymphopenia (586 cells/μL) and prolonged coagulation times (INR 1.62, aPTT 84.8 s). He had prerenal azotemia (serum creatinine of 2.94 mg/dL; BUN, 60.5 mg/dL) and uncompensated metabolic acidosis (pH of 7.29; pCO_2_, 36 mmHg; HCO_3_, 17.3 mmol/L; and lactate, 6.5 mmol/L). His liver function tests (LFTs) were the following: SGOT of 167 UI/L; SGPT, 60 UI/L; alkaline phosphatase, 168 UI/L; total bilirubin, 29.9 mg/dL (conjugated, 19.9 mg/dL); total proteins, 5.3 g/dL; serum albumin, 2.4 g/dL; globulin, 2.9 g/dL; and lactate dehydrogenase (LDH), 2488 UI/L. Viral markers for hepatitis B and C were all negative. Ultrasonography confirmed the diagnosis of hepatomegaly (18.5 cm) and also reported heterogeneous echogenicity (mimicking fatty infiltration) with a geographic pattern. Coagulopathy precluded a percutaneous biopsy. Sepsis was rationally ruled out given clinical and paraclinical considerations. He was admitted and**,** on his second inpatient day, he had hematuria and hematochezia without hemodynamic compromise. He was treated with fresh frozen plasma and intravenous vitamin K, stopping thereby the bleeding. 

On his fourth inpatient day, he presented progressive neurological deterioration leading to stupor, which was classified as grade 3 hepatic encephalopathy. Brain MRI ruled out hemorrhagic or infiltrative lesions, but demonstrated cerebral edema. Our patient went into a comatose state with hemodynamic compromise. 

**Figure 1:**
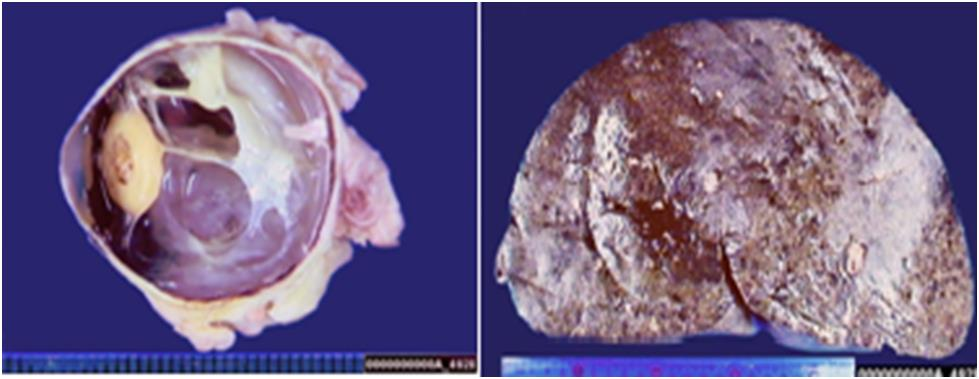
The sagittal section of the eye reveals an irregular pigmented lesion of the ciliary body (left panel). Gross appearance of the liver (right panel).

**Figure 2:**
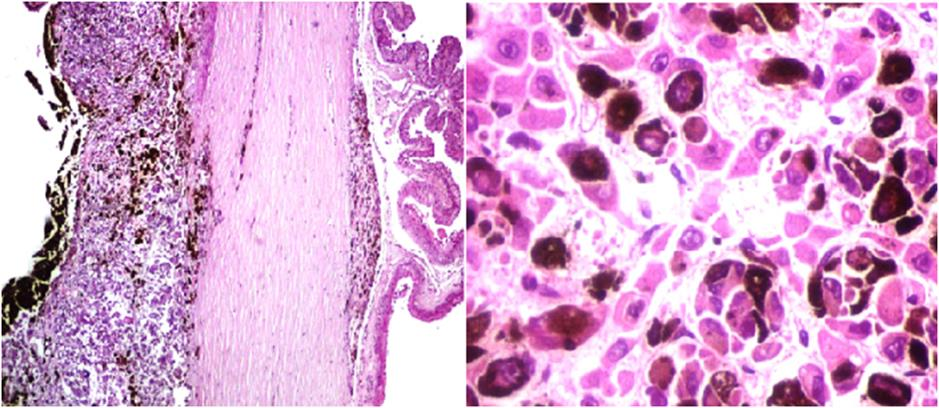
Pigmented cells with atypia and pleomorphism prominent ovoid nuclei with epithelioid features which originate in the choroid and focally infiltrate the sclera and ciliary body base (hematoxylin-eosin stain, left panel). Diffuse hepatic infiltration. Atypical pleomorphic cells with prominent ovoid nuclei and epithelioid features. Abundant pigment was evident in their cytoplasm and between hepatic sinusoids (right panel).

He developed sinus bradycardia, pulseless electrical activity and subsequently asystole. On post-mortem examination, pigmented cells with atypia and pleomorphism, prominent ovoid nuclei with epithelioid features, which originate in the choroid and focally infiltrate the sclera and ciliary body base**, **were seen. Hepatomegaly (8 kg) with neoplastic infiltration was also observed (figs. 1 and 2). 

## Discussion

The latter is a very uncommon presentation of this neoplasm. There are some reports describing the occurrence of ALF in the presence of other malignancies (7-10). However, ALF secondary to primary uveal melanoma infiltrating the liver is extremely rare (3-6). There are at least four previous cases reported in the literature (3-6). In 1992, Lesur et al. reported a patient with a rapid progressive liver failure treated for a choroidal melanoma 16 years earlier (4). Since then, three other reports of ALF in this setting have been described (5, 6). Except for the first three, all other cases were middle-aged adults (47-62 years) with an infiltrative pattern of liver injury in LFTs followed by an aggressive course with multiorgan involvement leading to death in several hours to few days. 

In adults, melanoma is the most common malignant primary intraocular tumor. It is usually located in the choroid (90%) or in the ciliary body and iris (5%). The probability of hematogenous dissemination is relatively high, the most affected site being the liver (>90%) (2). In general, its prognosis is poor with a reported median survival of 4-6 months and a 1-year survival of 10-15% (11). However, in the setting of ALF (as in our case), all patients died (3-6).

Although some therapeutic options to eradicate and prevent local recurrence of localized melanoma are available, no effective therapies for metastatic uveal melanoma currently exists (12).

 Kodjikan et al. described a cohort of 602 patients with uveal melanoma. Of these, 63 patients with liver metastasis were identified by screening ultrasound. They reported a median survival of 25 months after complete metastasectomy followed by postoperative transcatheter arterial infusion therapy (13). Nonetheless**, **as previously stated, the in-hospital mortality rate in ALF setting is extremely high. While there is no evidence that screening for metastatic disease improves the outcome in patients with uveal melanoma; it seems advisable to perform it in all affected patients due to its high rate of hematogenous spread (11).

Due to its unspecific presentation and inherent severity, the liver biopsy is of great importance for diagnosis. However, as in our case, this is not always possible because of bleeding and coagulopathy. Liver biopsy obtained by transjugular route may be a safer option (4). The histological pattern more frequently found in metastatic disease is the presence of cluster of nodules; although in the setting of ALF, massive diffuse infiltration of liver sinusoids is characteristic.

LDH surge has been proposed to be secondary to hepatic ischemic injury as it has been observed in lymphoma infiltrating the liver (14). This same mechanism was likely to be responsible for the fulminant liver failure in our patient. In the setting of malignant melanoma, this significant rise in LDH has been previously recognized as an ominous sign of ALF (15). In summary, liver metastasis should be kept in mind when assessing abnormal LFTs or ALF in patients with uveal malignant melanoma.
